# Two New Anisic Acid Derivatives from Endophytic Fungus *Rhizopycnis vagum* Nitaf22 and Their Antibacterial Activity

**DOI:** 10.3390/molecules23030591

**Published:** 2018-03-06

**Authors:** Ali Wang, Peng Li, Xuping Zhang, Peipei Han, Daowan Lai, Ligang Zhou

**Affiliations:** Department of Plant Pathology, College of Plant Protection, China Agricultural University, Beijing 100193, China; wangali526@163.com (A.W.); ytmuyue1989@163.com (P.L.); zhangxuping5@163.com (X.Z.); mhanpeipei@126.com (P.H.); dwlai@cau.edu.cn (D.L.)

**Keywords:** endophytic fungus, *Rhizopycnis vagum*, anisic acid derivatives, rhizopycnis acids A and B, antibacterial activity

## Abstract

Rhizopycnis acids A (**1**) and B (**2**), two new anisic acid derivatives, were obtained from the ethyl acetate extract of the fermentation cultures of *Rhizopycnis vagum*, an endophytic fungus isolated from the healthy tissues of *Nicotiana tabacum*. The structures of the two compounds were determined through a series of 1D and 2D NMR and HRMS spectral analyses. Both compounds were the first anisic acid derivatives containing methylbutanoic/methylbutenoic acid group found in fungi. **1** and **2** displayed antibacterial activity against six tested bacteria with IC_50_ values in the range 16.1~81.3 μg/mL.

## 1. Introduction

Endophytic fungi are a rich source of secondary metabolites with unique structures, exhibiting a wide range of biological activities with potential applications in agriculture, medicine and food industry [1,2,3,4]. The fungus *Rhizopycnis vagum* has been reported as an endophyte in *Dioscorea zingiberensis* [5], *Juniperus communis* [6], *Nicotiana tabacum* [7] and *Zingiber officinale* [8].

**Figure 1:**
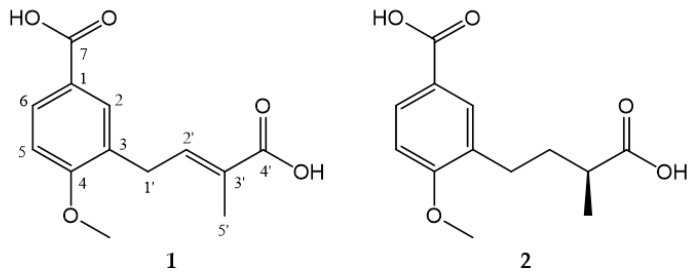
Structures of the compounds **1** and **2**.

Chemical and biological investigation for the endophytic fungus *Rhizopycnis vagum* Nitaf22 from *N. tabacum*, have led to the isolation of bioactive dibenzo-α-pyrone derivatives [7]. In our ongoing search for new bioactive metabolites from this fungus, two new anisic acid derivatives containing methylbutanoic or methylbutenoic acid group (Figure 1) were isolated. They were screened for antibacterial activities. Herein, we reported the isolation, structural elucidation, and antibacterial activity of these two compounds.

## 2. Results and Discussion

### 2.1. Structural Elucidation of the Compounds

Compounds **1** and **2** (Figure 1) were purified from EtOAc extract of *R. vagum* fermentation cultures through vacuum liquid chromatography, MPLC over silica gel, Sephadex LH-20, and reverse-phase semi-preparative HPLC.

**Figure 2:**
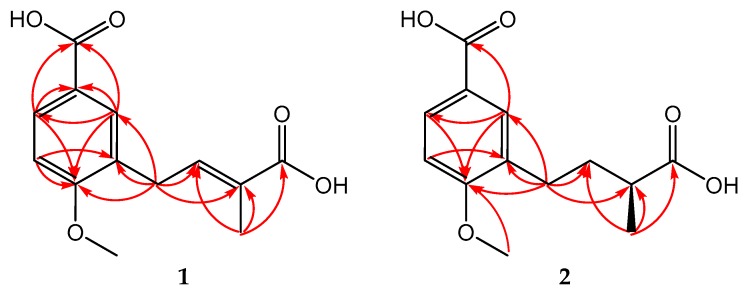
Key HMBC (H→C) correlations of the compounds **1** and **2**.

Compound **1** was obtained as a light yellow amorphous powder. It had the molecular formula C_13_H_14_O_5_ (seven degrees of unsaturation), based on analysis of its HRESIMS at *m*/*z* 249.0771 [M − H]^−^ (calcd for C_13_H_13_O_5_, 249.0768) (Figure S1). The IR spectrum showed absorption for hydroxyl at 3500 cm^−1^, intense and sharp absorption for carbonyl at 1681 cm^−1^, and absorptions for phenyl at 1427, 1503 and 1604 cm^−1^ (Figure S2). The ^1^H NMR spectrum (Table 1) displayed the signals of 1,3,4-trisubstituted aromatic system at *δ*_H_ 7.93 (d, *J* = 8.6 Hz), 7.80 (s) and 7.03 (d, *J* = 8.6 Hz), an olefinic signal at *δ*_H_ 6.85 (t, *J* = 7.5 Hz), a methoxy signal at *δ*_H_ 3.92, a methene signal at *δ*_H_ 3.53 (d, *J* = 7.5 Hz), and a singlet methyl single at *δ*_H_ 1.93 (Figure S3). Corresponding to ^1^H NMR spectrum, the ^13^C NMR spectrum (Table 1) displayed 13 carbon signals, including two carbonyls (*δ*_C_ 171.6, 169.9), eight sp^2^ carbons (*δ*_C_ 162.7, 141.1, 132.3, 131.6, 129.7, 128.7, 124.0, 111.1), one methoxy carbon (*δ*_C_ 56.3), and two aliphatic carbons (*δ*_C_ 30.4, 12.5) (Figure S4). The placement of the above groups was confirmed by analysis of HMBC spectrum (Figure S5). The correlations from H-2 to C-1, C-4, and C-6; from H-5 to C-3 and C-4; and from H-6 to C-1 and C-4, verified the presence of 1,3,4-trisubstituted aromatic system. Furthermore, a carboxyl group was attached to C-1 by the correlations from H-2/H-6 to C-7. Based on the chemical shift of C-4 (*δ*_C_ 162.7) and 4-OCH_3_ (*δ*_H_, _C_ 3.92, 56.3), and NOESY correlation between 4-OCH_3_ and H-5, the methoxy was linked to C-4 (Figure S6). These correlations established a 3-substituted anisic acid skeleton of **1**. The HMBC correlations from H_2_-1′ to C-2′ and C-3′ and from H_3_-5′ to C-2′, C-3′ and C-4′, suggested a methylbutenoic acid substituent. The HMBC correlations from H_2_-1′ to C-2, C-3 and C-4 confirmed this substituent being located at C-3 of the aromatic ring (Figure 2). The planar structure of **1** was then determined as shown in Figure 1. The *E* configuration of the double bond in the side chain was concluded from the NOESY correlation between H_3_-5′ and H_2_-1′. Therefore, the structure of **1** was elucidated as (*E*)-3-(3-carboxybut-2-en-1-yl)-4-methoxybenzoic acid, which was named as rhizopycnis acid A.

Compound **2** was isolated as a yellow amorphous powder, and has the molecular formula C_13_H_16_O_5_ (six degrees of unsaturation), as deduced from the HRESIMS at *m*/*z* 251.0930 [M − H]^−^ (calcd for C_13_H_15_O_5_, 251.0925) (Figure S7). The IR spectrum showed similar absorptions to those attributed to the hydroxyl (3500 cm^−1^), carbonyl (1681 cm^−1^), and phenyl (1443, 1505, 1606 cm^−1^) groups found in **1** (Figure S8). The NMR spectral data of **2** are also similar to those of **1**, but differed in the side chain (Figures S9 and S10). The signals for the double bond in **1** were replaced by one methylene (*δ*_C_, 34.9) and methine (*δ*_C_, 40.5) in **2**. In addition, the methyl group was shifted upfield and appeared as a doublet at *δ*_H_ 1.18 (d, *J* = 7.0 Hz). These suggested **2** was a C2′/C3′ saturated derivative of **1**. This was corroborated by analysis of the HMBC spectrum, in which correlations were observed from H_2_-1′ to C-2′ and C-3′ and from H_3_-5′ to C-2′, C-3′ and C-4′ (Figure 2 and Figure S11). Thus, the planar structure of **2** was determined as shown in Figure 1. By comparing the optical rotation value {[α${]}_{D}^{25}$ +2.67 (c 0.15, MeOH)} of **2** with (*S*)-2-methyl-3-phenylpropionic acid {[α${]}_{D}^{22}$ +26.3 (c 1.0, CHCl_3_)} and (*R*)-2-methyl-3-phenylpropionic acid {[α${]}_{D}^{22}$ −18 (c 1.0, CHCl_3_)} [9], the absolute configuration of **2** was proposed to be *S*. Other examples related to the optical rotation values included (*R*)-2-methyl-4-phenylbutanoic acid ([α${]}_{D}^{25}$ −28.0) [10] as well as 2(*S*)-methylbutyric acid ([α]_D_ +19) and 2(*R*)-methylbutyric acid ([α]_D_ −19) [11]. Therefore, the structure of **2** was elucidated as (*S*)-3-(3-carboxybutyl)-4-methoxybenzoic acid, which was named as rhizopycnis acid B.

The structures of **1** and **2** incorporated methylbutanoic/methylbutenoic acid and anisic acid moieties. A similar compound, 3-(3-methylbut-2-en-1-yl)-4-methoxybenzoic acid, was previously isolated from the stems of a perennial plant *Wigandia urens* (Hydrophyllaceae) [12]. The differences were ascribed to a different side chain locating at C-3 (Figure 1). To the best of our knowledge, these three compounds were the only anisic acid derivatives containing an isopentyl group in natural products. The anisic acids with their structures containing methylbutanoic or methylbutenoic acid group (**1** and **2**) represent a new class of natural products found in fungi.

### 2.2. Antibacterial Activity

Both compounds were tested for their antibacterial activity against six pathogenic bacteria. Compounds **1** and **2** were active against all the tested bacteria (Table 2), and **1** showed stronger inhibition. This suggested that the C-2′/3′ olefinic bond positively correlated to the antibacterial activity. To our knowledge, this is the first report of antibacterial activity for this type of natural product. Though these two compounds are not as active as the positive control streptomycin sulfate, they show potential as the antibacterial agents. The similar compound 3-(3-methylbut-2-en-1-yl)-4-methoxybenzoic acid was reported to exhibit anti-HIV activity against CCR5 receptor with IC_50_ value of 26 μM [12]. Therefore, other biological activities, including the anti-HIV activity of compounds **1** and **2**, are necessary to study.

## 3. Materials and Methods

### 3.1. General Experimental Procedures

Optical rotations were measured using Autopol VI automatic polarimeter (Rudolph Research Analytical, Hackettstown, NJ, USA). Ultraviolet (UV) spectra were scanned by a TU-1810 UV−VIS spectrophotometer (Beijing Persee General Instrument Co., Ltd., Beijing, China). Infrared (IR) spectra were measured on a Thermo Scientific Nicolet iS50 FT-IR spectrometer (Thermo Fisher Scientific Inc., Madison, WI, USA). High-resolution electrospray ionization mass spectrometry (HRESIMS) spectra were obtained by LC 1260-Q-TOF/MS 6520 machine (Agilent Technologies, Santa Clara, CA, USA). ^1^H, ^13^C, HMBC and NOESY spectra were acquired on an Avance 400 NMR spectrometer (Bruker BioSpin, Zurich, Switzerland). Chemical shifts were expressed in *δ* (ppm) referring to the inner standard tetramethylsilane, and coupling constants (*J*) in hertz (Hz). Silica gel (200~300 mesh, Qingdao Marine Chemical Inc., Qingdao, China) and Sephadex LH-20 (Pharmacia Biotech, Uppsala, Sweden) were used for column chromatography. Medium-pressure liquid chromatography (MPLC) separation was carried out on an Eyela-VSP-3050 instrument (Tokyo Rikakikai Co., Tokyo, Japan). High-performance liquid chromatography (HPLC) was performed with a Shimadzu LC-20A instrument employing an SPD-M20A photodiode array detector (Shimadzu Corp., Tokyo, Japan), and an analytic C_18_ column (250 mm × 4.6 mm i.d., 5 μm; Phenomenex Inc., Torrance, CA, USA) was used for HPLC separation. Semi-preparative HPLC separation was carried out on a Lumtech K-501 pump (Lumiere Tech. Ltd., Beijing, China) with a K-2501 UV detector using a Luna-C18 column (250 mm × 10 mm i.d., 5 μm, Phenomenex Inc., Torrance, CA, USA). Thin-layer chromatography (TLC) was carried out on glass precoated silica gel GF254 plates (Qingdao Marine Chemical Inc.). Spots were visualized under UV light (254 or 365 nm) or by spraying with 10% H_2_SO_4_ in 95% EtOH followed by heating at 100 °C.

### 3.2. Fungal Material and Fermentation

The endophytic fungus *Rhizopycnis vagum* Nitaf22 has been previously isolated from the inner tissue of the healthy root of a three-year-old *Nicotiana tabacum* [7]. Its gene sequence was submitted to GenBank database to obtain the accession number KM095527, and the pure cultures were deposited at the Department of Plant Pathology, China Agricultural University, Beijing, China.

The fungus was cultured as the reported procedure [7], while the rescale-up fermentation was carried out by using a total of 10 kg of rice as the solid medium under static conditions at room temperature (RT) in the dark for 50 days.

### 3.3. Extraction and Isolation

The fermented rice was chopped and then extracted with EtOAc at RT three times. After filtration, the solvent was removed under vacuum on a rotatory evaporator to yield 163 g of a brownish residue. The crude extract was partitioned through vacuum liquid chromatography (column diameter 8 cm; height 10 cm) by eluting first with a gradient of acetone in petroleum ether and subsequently with a gradient of MeOH in CH_2_Cl_2_ to obtain five fractions. The second subfraction (Fr. B 16.8 g) was applied to MPLC (column diameter at 3 cm; height at 50 cm) over silica gel and eluted with a gradient of MeOH in CH_2_Cl_2_ to afford six fractions (Frs. B1~B6). Fr. B5 was separated by Sephadex LH-20 gel-permeation chromatography (column diameter at 3 cm; height at 60 cm) using petroleum ether/CH_2_Cl_2_/MeOH (5:5:1, *v*/*v*/*v*) as an eluent to give Frs. B5-1~B5-4. Fr. B5-3 was further purified by reverse-phase semi-preparative HPLC with MeOH/H_2_O/TFA (60:40:0.02, *v*/*v*/*v*) to yield pure compounds **1** (5.5 mg) and **2** (2.2 mg).

Rhizopycnis acid A ((*E*)-3-(3-carboxybut-2-en-1-yl)-4-methoxybenzoic acid; **1**): light yellow, amorphous solid; UV (MeOH) λ_max_ (log ε) 218 (4.65), 253 (4.44) nm; IR ν_max_ 3714, 3648, 3614, 2919, 2849, 2651, 1681, 1645, 1604, 1503, 1427, 1336, 1298, 1256, 1196, 1136, 1105, 1023, 930, 902, 830, 774, 737, 648, 631, 563, 450, 418 cm^−1^; HRESIMS *m*/*z* 249.0771 [M − H]^−^ (calcd for C_13_H_13_O_5_, 249.0768); ^1^H NMR and ^13^C NMR data are shown in Table 1.

Rhizopycnis acid B ((*S*)-3-(3-carboxybutyl)-4-methoxybenzoic acid; **2**): yellow, amorphous solid; [α${]}_{D}^{25}$ +2.67 (c 0.15, MeOH); UV (MeOH) λ_max_ (log ε) 210 (4.00), 253 (3.86) nm; IR ν_max_ 3714, 3648, 3614, 2920, 2850, 1868, 1681, 1648, 1606, 1505, 1443, 1262, 1190, 1139, 1026, 904, 841, 800, 776, 724, 627, 576, 523, 419 cm^−1^; HRESIMS *m*/*z* 251.0930 [M − H]^−^ (calcd for C_13_H_15_O_5_, 251.0925); ^1^H NMR and ^13^C NMR data are shown in Table 1.

### 3.4. Antibacterial Activity Assay

The antibacterial activities of compounds **1** and **2** were tested against six bacterial strains *Bacillus subtilis* ATCC 11562 (G^+^), *Staphylococcus hemolyticus* ATCC 29970 (G^+^), *Agrobacterium tumefaciens* ATCC 11158 (G^−^), *Pseudomonas lachrymans* ATCC 11921 (G^−^), *Ralstonia solanacearum* ATCC11696 (G^−^) and *Xanthomonas vesicatoria* ATCC 11633 (G^−^). All these test bacteria were deposited at the College of Plant Protection, China Agricultural University, Beijing, China. According to our reported approach, a modification of the broth dilution microplate MTT colorimetric test was used to detect the activity [13,14]. 30% DMSO without compounds was used as the negative control, and streptomycin sulfate was used as the positive control. The measuring of samples was performed in triplicate.

## 4. Conclusions

In this study, two new anisic acid derivatives namely rhizopycnis acids A (**1**) and B (**2**) were isolated from the endophytic fungus *R. vagum* obtained from *N. tabacum*. Their structures were determined through a series of 1D and 2D NMR and HRMS spectral analyses. Both compounds were the first fungal anisic acid derivatives with their structures containing methylbutanoic/methylbutenoic acid group. They exhibited moderate antibacterial activity, which showed their potential as the antibacterial agents.

## Figures and Tables

**Table 1:** ^1^H NMR (400 MHz) and ^13^C NMR (100 MHz) data of compounds **1** and **2** in CD_3_OD.

Position	1		2	
	*δ*_H_, Mult. (*J* in Hz)	*δ*_C_, Type	*δ*_H_, Mult. (*J* in Hz)	*δ*_C_, Type
1		111.1, C		110.9, C
2	7.80, s	132.3, CH	7.79, s	132.4, CH
3		128.7, C		131.3, C
4		162.7, C		162.8, C
5	7.03, d (8.6)	124.0, CH	6.98, d (8.6)	124.0, CH
6	7.93, d (8.6)	131.6, CH	7.89, d (8.6)	131.0, CH
7		169.9, C		170.0, C
4-OCH_3_	3.92, s	56.3, CH_3_	3.89, s	56.1, CH_3_
1′	3.53, d (7.5)	30.4, CH_2_	2.66, t (7.2)	28.8, CH_2_
2′	6.85, t (7.5)	129.7, CH	1.66, m1.92, m	34.9, CH_2_
3′		141.1, C	2.40, m	40.5, CH
4′		171.6, C		180.7, C
5′	1.93, s	12.5, CH_3_	1.18, d (7.0)	17.7, CH_3_

**Table 2:** Antibacterial activity of compounds **1** and **2**.

Bacterium	MIC (IC_50_) (μg/mL)		
	1	2	Streptomycin Sulfate (CK^+^)
*A. tumefaciens*	64 (20.82 ± 0.42)	128 (70.89 ± 1.02)	16 (3.82 ± 0.34)
*B. subtilis*	64 (16.11 ± 1.23)	128 (81.28 ± 0.99)	16 (6.11 ± 0.04)
*P. lachrymans*	64 (23.48 ± 0.74)	64 (21.23 ± 1.28)	16 (4.16 ± 0.60)
*R. solanacearum*	64 (29.46 ± 0.37)	128 (43.40 ± 1.53)	16 (3.29 ± 0.06)
*S. hemolyticus*	64 (21.11 ± 0.98)	128 (67.61 ± 1.59)	16 (8.97 ± 0.98)
*X. vesicatoria*	64 (24.31 ± 0.89)	128 (34.86 ± 1.51)	16 (6.10 ± 0.50)

## Supplementary Materials

Supplementary materials (Figures S1–S11) associated with this article, including HRESIMS, IR, 1D and 2D NMR spectra of **1** and **2** can be found in the online version.

## References

[1] Zhao J., Shan T., Mou Y., Zhou L. (2011). Plant-derived bioactive compounds produced by endophytic fungi. Mini-Rev. Med. Chem..

[2] Nisa H., Kamili A.N., Nawchoo I.A., Shafi S., Shameem N., Bandh S.A. (2015). Fungal endophytes as prolific source of phytochemicals and other bioactive natural products: A review. Microb. Pathog..

[3] Deepika V.B., Murali T.S., Satyamoorthy K. (2016). Modulation of genetic clusters for synthesis of bioactive molecules in fungal endophytes: A review. Microbiol. Res..

[4] Martinez-Klimova E., Rodriguez-Pena K., Sanchez S. (2017). Endophytes as sources of antibiotics. Biochem. Pharmacol..

[5] Xu L., Zhou L., Zhao J., Li J., Li X., Wang J. (2008). Fungal endophytes from *Dioscorea zingiberensis* rhizomes and their antibacterial activity. Lett. Appl. Microbiol..

[6] Knapp D.G., Pintye A., Kovacs G.M. (2012). The dark side is not fastidious-dark septate endophytic fungi of native and invasive plants of semiarid sandy areas. PLoS ONE.

[7] Lai D., Wang A., Cao Y., Zhou K., Mao Z., Dong X., Tian J., Xu D., Dai J., Peng Y. (2016). Bioactive dibenzo-α-pyrone derivatives from the endophytic fungus *Rhizopycnis vagum* Nitaf22. J. Nat. Prod..

[8] Ginting R.C.B., Sukarno N., Widyastuti U., Darusman L.K., Kanaya S. (2013). Diversity of endophytic fungi from red ginger (*Zingiber officinale* Rosc.) plant and their inhibitory effect to *Fusarium oxysporum* plant pathogenic fungi. HAYATI J. Biosci..

[9] Ueberbacher B.J., Griengl H., Weber H. (2008). Chemo-enzymatic synthesis of new ferrocenyl-oxazolidinones and their application as chiral auxiliaries. Tetrahedron: Asymmetry.

[10] Scrivanti A., Bovo S., Ciappa A., Matteoli U. (2006). The asymmetric hydrogenation of 2-phenethylacrylic acid as the key step for the enantioselective synthesis of Citralis Nitrile. Tetrahedron Lett..

[11] Cassani F., Celentano G., Erba E., Pocar D. (2004). New synthesis of optically pure α-branched aliphatic carboxylic acids from amidines. Synthesis.

[12] Cao S., Rossant C., Ng S., Buss A.D., Butler M.S. (2003). Phenolic derivatives from *Wigandia urens* with weak activity against the chemokine receptor CCR5. Phytochemistry.

[13] Lu S., Sun W., Meng J., Wang A., Wang X., Tian J., Fu X., Dai J., Liu Y., Lai D. (2015). Bioactive bis-naphtho-γ-pyrones from rice false smut pathogen *Ustilaginoidea virens*. J. Agric. Food Chem..

[14] Xu L., Wang X., Luo R., Lu S., Guo Z., Wang M., Liu Y., Zhou L. (2015). Secondary metabolites of rice sheath blight pathogen *Rhizoctonia solani* Kühn and their biological activities. J. Integr. Agric..

