# Supplementary material for: Two New Anisic Acid Derivatives from Endophytic Fungus Rhizopycnis vagum Nitaf22 and Their Antibacterial Activity
Source: Molecules. 2018 Mar 6;23(3):591. doi: 10.3390/molecules23030591 (PMC6017559; doi:10.3390/molecules23030591)
Supplement: Supplementary file 1 [file molecules-23-00591-s001.pdf]

*Supplementary Materials*

# Two New Anisic Acid Derivatives from Endophytic fungus *Rhizopycnis vagum* Nitaf22 and Their Antibacterial activity

**Ali Wang, Peng Li, Xuping Zhang, Peipei Han, Daowan Lai and Ligang Zhou ***

Department of Plant Pathology, College of Plant Protection, China Agricultural University, Beijing 100193, China; wangali526@163.com (A.W.); ytmuyue1989@163.com (P.L.); zhangxuping5@163.com (X.Z.); mhanpeipei@126.com (P.H.); dwlai@cau.edu.cn (D.L.)

* Correspondence: lgzhou@cau.edu.cn; Tel.: +86-10-6273-1199

## Contents

**Figure S1.** HRESIMS spectrum of **1**. ........ 2

**Figure S2.** IR spectrum of **1**........ 2

**Figure S3.** $^1$H NMR spectrum of **1** ($CD_3OD$, 400 MHz)........ 3

**Figure S4.** $^{13}$C NMR spectrum of **1** ($CD_3OD$, 100 MHz)........ 3

**Figure S5.** HMBC spectrum of **1** (400 MHz). ........ 4

**Figure S6.** NOESY spectrum of **1** (400 MHz). ........ 4

**Figure S7.** HRESIMS spectrum of **2**. ........ 5

**Figure S8.** IR spectrum of **2**........ 5

**Figure S9.** $^1$H NMR spectrum of **2** ($CD_3OD$, 400 MHz)........ 6

**Figure S10.** $^{13}$C NMR spectrum of **2** ($CD_3OD$, 100 MHz)........ 6

**Figure S11.** HMBC spectrum of **2** (400 MHz). ........ 7

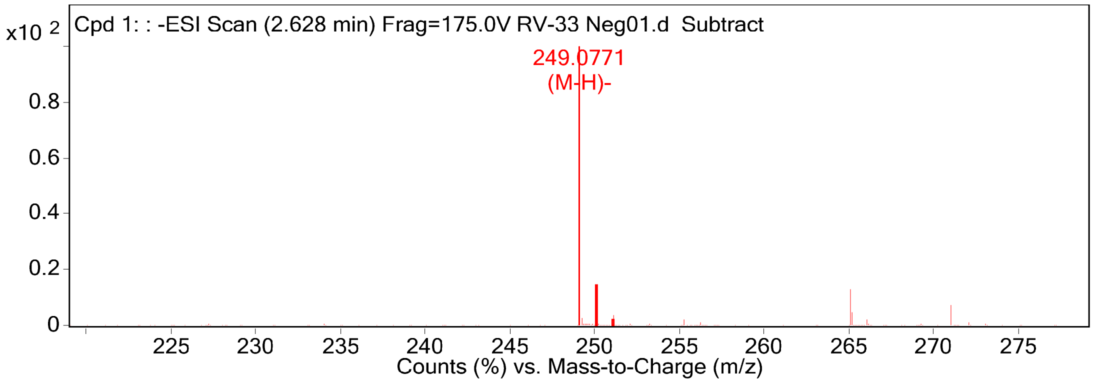


| *m/z* | *Calc m/z* | Diff (ppm) | z | Abund | Formula | Ion |
|---|---|---|---|---|---|---|
| 249.0771 | 249.0768 | 1.14 | -1 | 329658.7 | $C_{13}H_{13}O_5$ | $(M-H)^-$ |

**Figure S1.** HRESIMS spectrum of **1**.

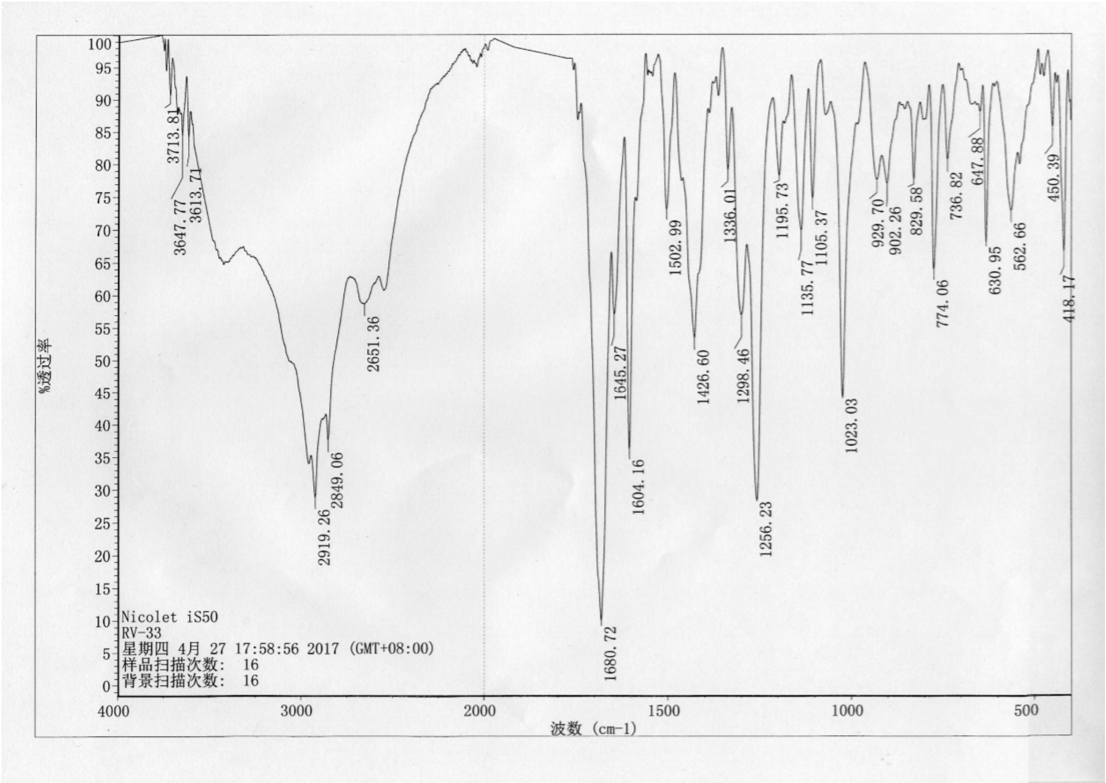


**Figure S2.** IR spectrum of **1**.

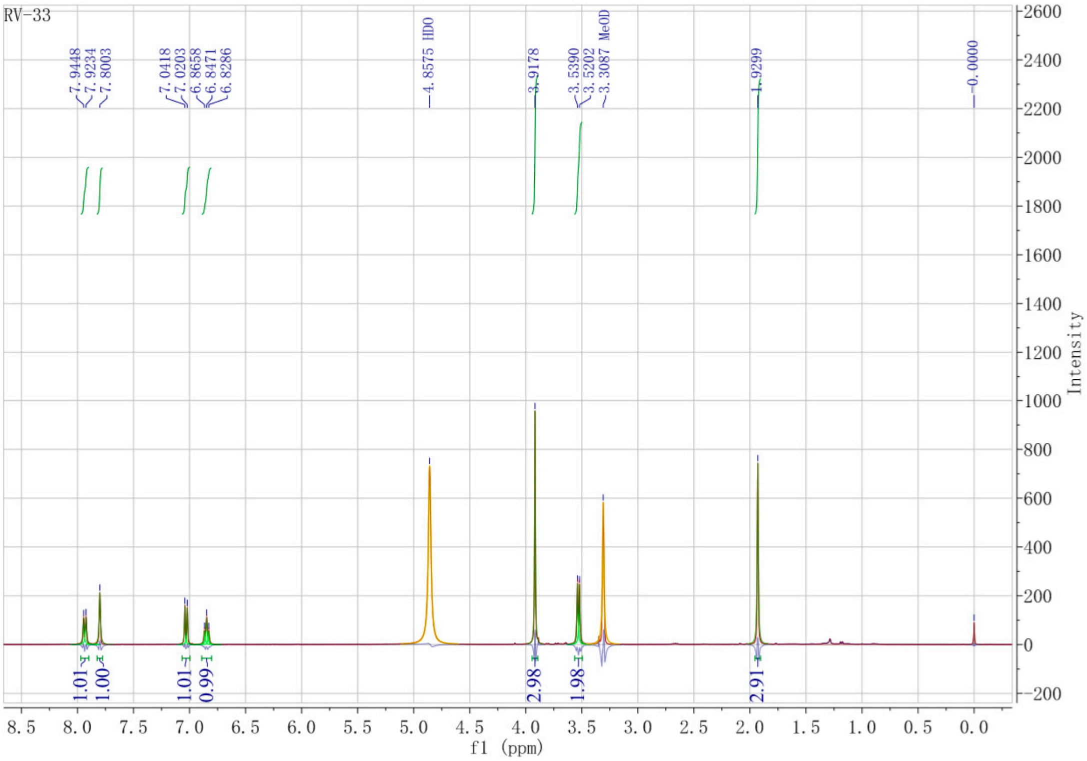


**Figure S3.** $^{1}$H NMR spectrum of **1** ($CD_3OD$, 400 MHz).

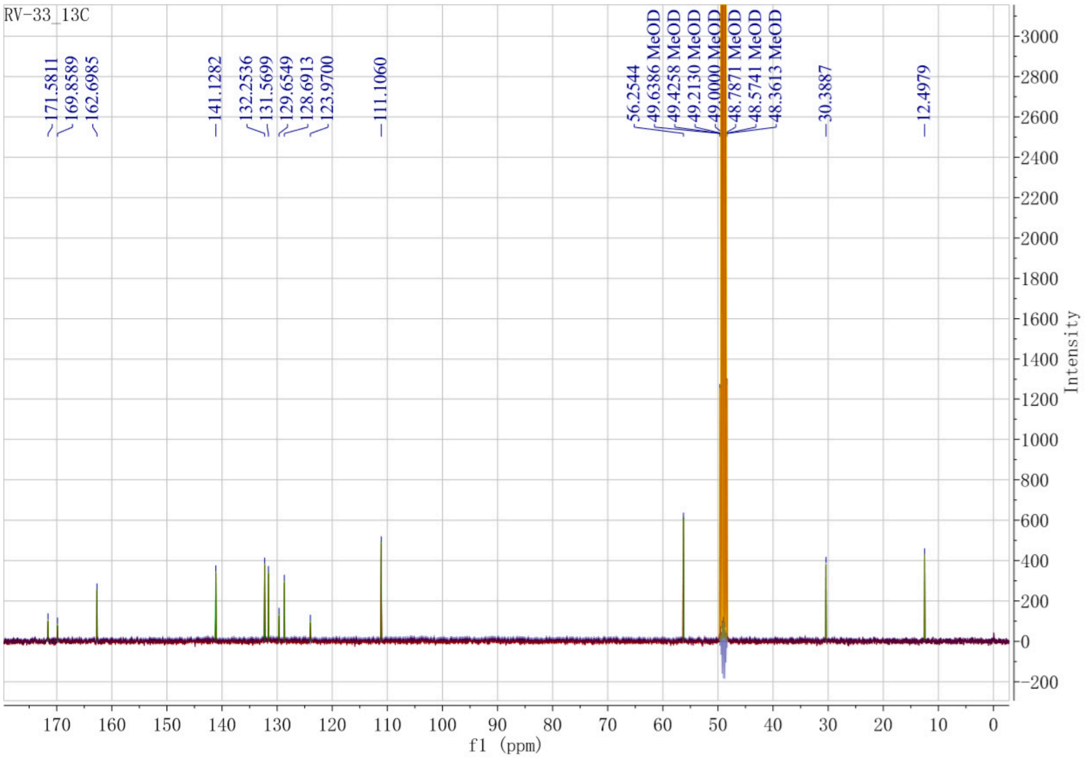


**Figure S4.** $^{13}$C NMR spectrum of **1** ($CD_3OD$, 100 MHz).

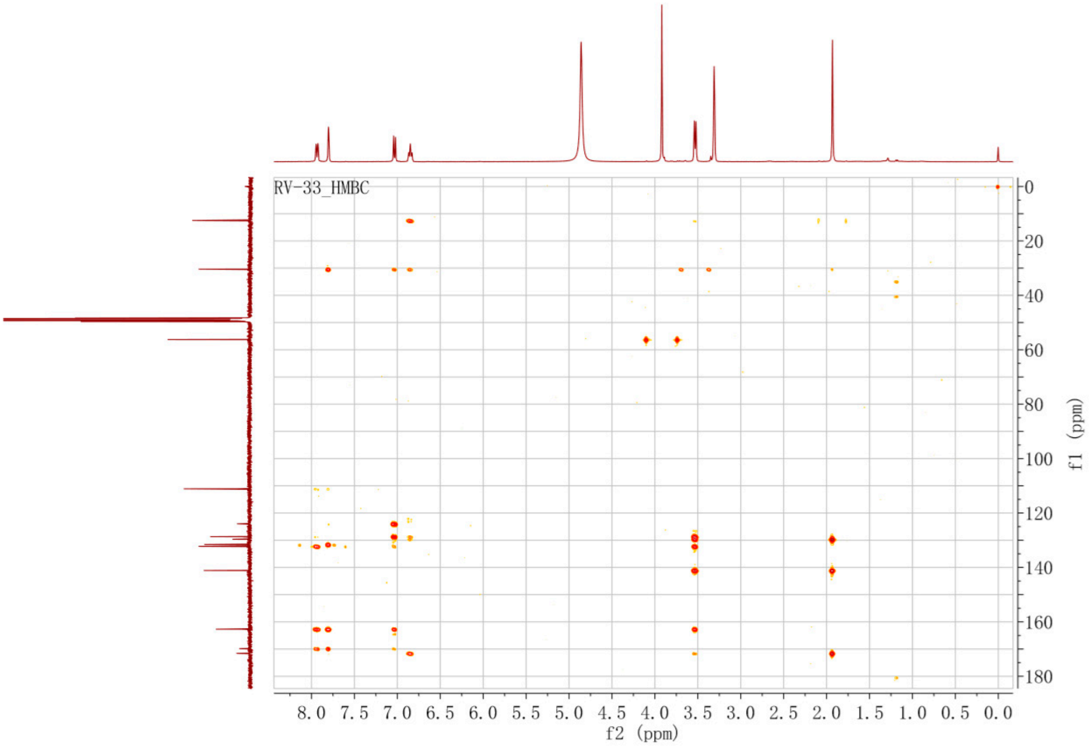


**Figure S5.** HMBC spectrum of **1** (400 MHz).

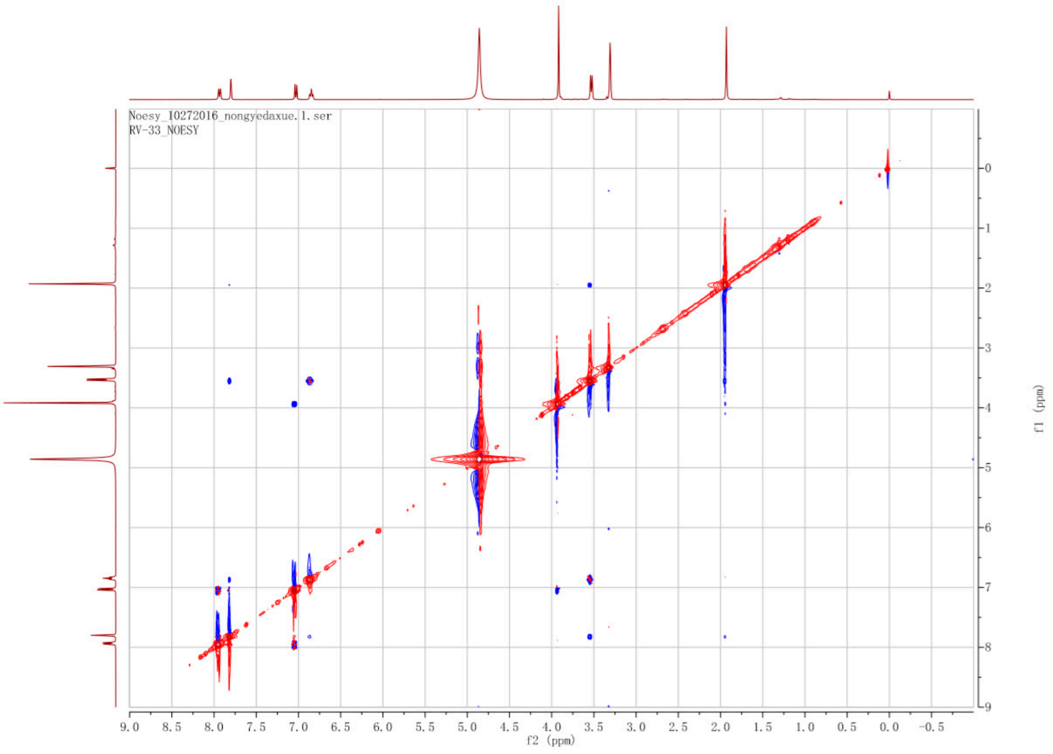


**Figure S6.** NOESY spectrum of **1** (400 MHz).

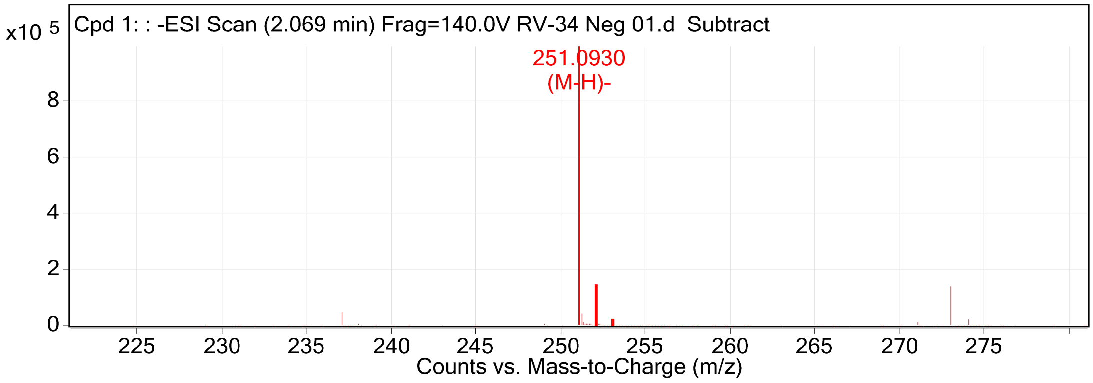


| *m/z* | *Calc m/z* | Diff (ppm) | z | Abund | Formula | Ion |
|---|---|---|---|---|---|---|
| 251.093 | 251.0925 | 2.01 | -1 | 991886.9 | $C_{13}H_{15}O_5$ | $(M-H)^-$ |

**Figure S7.** HRESIMS spectrum of **2**.

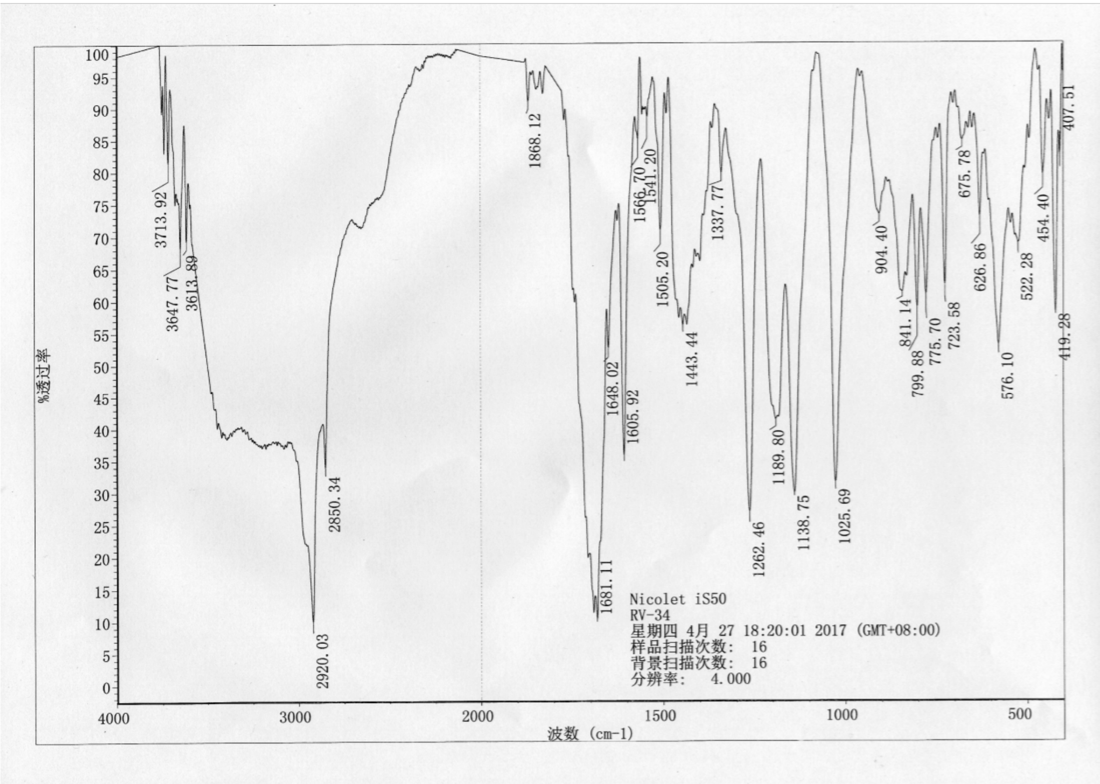


**Figure S8.** IR spectrum of **2**.

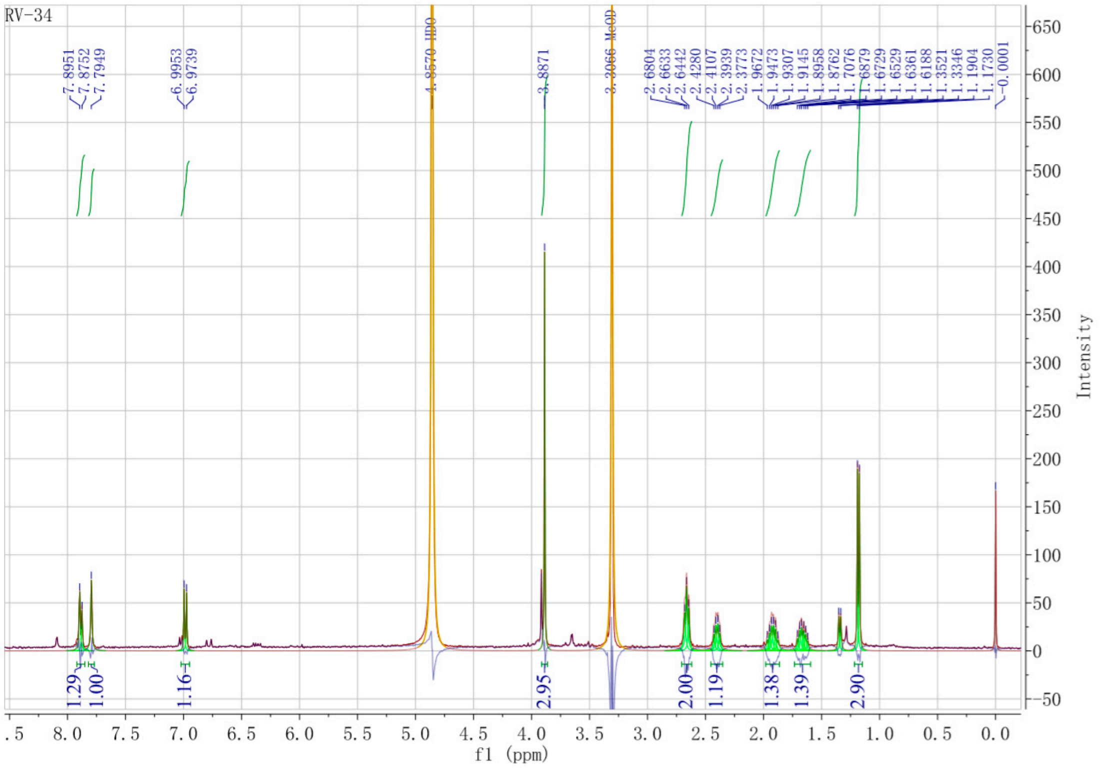


**Figure S9.** $^{1}H$ NMR spectrum of **2** ($CD_3OD$, 400 MHz).

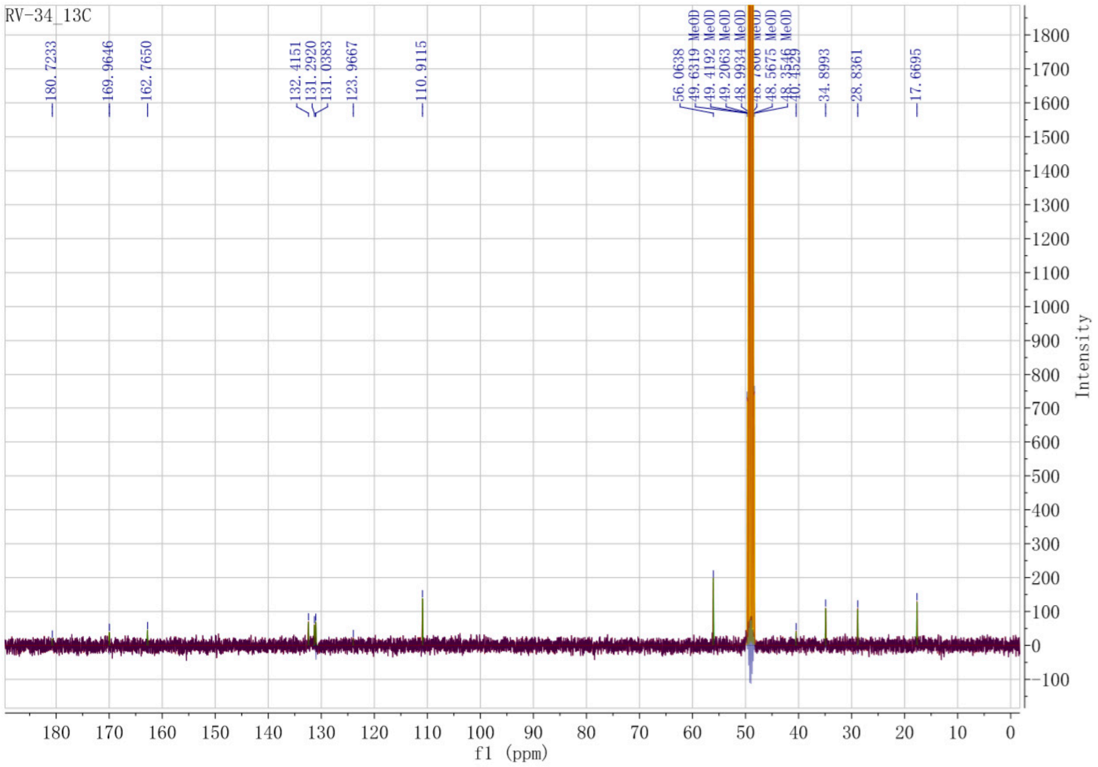


**Figure S10.** $^{13}C$ NMR spectrum of **2** ($CD_3OD$, 100 MHz).

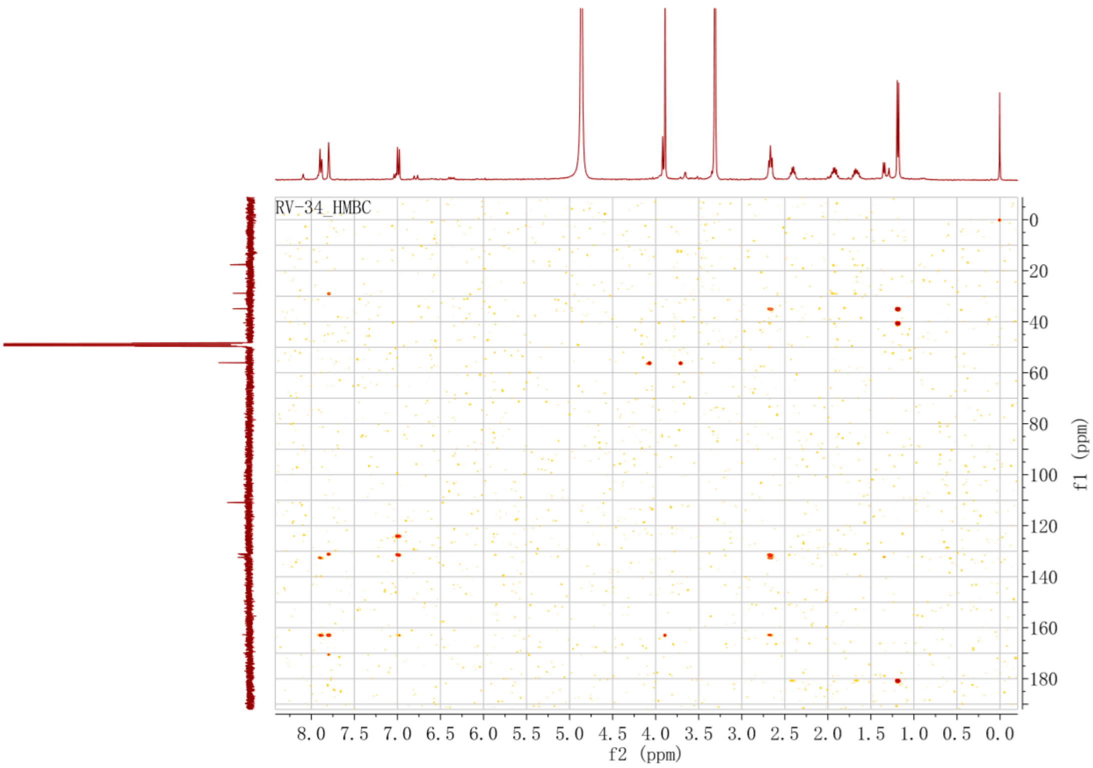


**Figure S11.** HMBC spectrum of **2** (400 MHz).
